# Risk factors for delayed antiretroviral therapy initiation among HIV-seropositive patients

**DOI:** 10.1371/journal.pone.0180843

**Published:** 2017-07-10

**Authors:** Terra V. Fatukasi, Stephen R. Cole, Richard D. Moore, William C. Mathews, Jessie K. Edwards, Joseph J. Eron, for the CNICS investigators

**Affiliations:** 1 Department of Epidemiology, University of North Carolina at Chapel Hill, Chapel Hill, North Carolina, United States of America; 2 School of Medicine, Johns Hopkins University, Baltimore, Maryland, United States of America; 3 School of Medicine, University of California San Diego, San Diego, California, United States of America; 4 Department of Medicine, Division of Infectious Diseases, University of North Carolina at Chapel Hill, Chapel Hill, North Carolina, United States of America; National and Kapodistrian University of Athens, GREECE

## Abstract

Prompt initiation of combination antiretroviral therapy (ART) is important to reduce comorbidity and mortality among people living with HIV, especially for those with a low CD4 cell count. However there is evidence that not everyone receives prompt initiation of ART after enrolling into HIV care. The current study investigated factors associated with failure to initiate ART within two years of entering into care among those with a CD4 count at or below 350 cells/mm^3^. The sample included 4,907 ART-naive patients with a CD4 count at or below 350 cells/mm^3^ enrolled between January 1, 2003 and December 31, 2012 at any of eight clinical sites in the Center for AIDS Research Network of Integrated Clinical Systems (CNICS). The two-year risk of delayed ART initiation was estimated using a log-binomial regression model with stabilized inverse probability of censoring weights for those lost to follow-up. Adjusting for other factors, an earlier enrollment date was the sole demographic characteristic associated with an increased risk of delayed ART initiation. Higher CD4 count, lower viral load, and a prevalent AIDS diagnosis were clinical characteristics associated with delayed ART initiation. Gender, age, race/ethnicity and HIV risk factors such as reported male-to-male sexual contact and injection drug use were not associated with delayed ART initiation. This study identified characteristics of patients for whom treatment was strongly to moderately recommended but who did not initiate ART within two years of entering care. Despite the known benefits of early antiretroviral therapy initiation, a lower viral load measurement may continue to be an important clinical characteristic in the more recent era with current ART initiation guidelines. These findings provide a target for closer monitoring and intervention to reduce disparities in HIV care.

## Introduction

Despite the introduction of effective combination antiretroviral therapy (ART) for HIV in 1996 [1], HIV continues to be a global health crisis. For example, in 2014, the United States Centers for Disease Control and Prevention (CDC) estimated 1.2 million US persons living with HIV and 12,333 AIDS-related deaths [2]. The use of ART has led to an increase in life expectancy among HIV-seropositive individuals through a reduction in HIV-associated comorbidity, as well as non-HIV and non-AIDS comorbidities [3–6]. The difference in life expectancy between HIV-seropositive and HIV-seronegative individuals is shrinking, though specific populations still exhibit notable differences [3, 6]. For example, among HIV-seropositive patients on ART, those with a history of injection drug use, a lower baseline CD4 count, and who are non-white have lower life expectancies relative to other groups [3, 6]

The current guidelines provided by the US Department of Health and Human Services (DHHS) recommend that ART be provided to all HIV-seropositive individuals to reduce disease progression and prevent HIV transmission [5]. While several studies have assessed characteristics associated with a faster time to ART initiation using time-to event analyses, we know of no other studies assessing the proportion of patients promptly initiating ART and their characteristics, especially among those for whom treatment was strongly to moderately recommended [7–10]. Some characteristics associated with a slower time to ART initiation include black non-Hispanic race/ethnicity, injection drug use, a greater number of psychosocial barriers, female gender, reporting male-to-male sexual contact, lack of health insurance, higher CD4 count, and lack of comorbidity [7–10]. A prior study among participants in the HIV Outpatient Study found that 20% of HIV-seropositive patients did not initiate ART within a 13.5-year study period from 2000–2013 [10]. Evidence from other countries looking at the proportion of patients initiating ART and their characteristics also suggests that not all HIV-seropositive individuals obtain prompt initiation of ART, even among those who have entered care [11,12]. It is unclear whether these same characteristics associated with a faster time to ART initiation among all HIV-seropositive patients are consistent characteristics of delayed ART initiation among those recommended for treatment. Pinpointing characteristics of those patients indicated for ART initiation, but who have not initiated ART, is especially relevant given current clinical guidelines that recommend treatment for all HIV-seropositive patients. A prior study using a clinic-based cohort in Uganda found that 18% of HIV-seropositive patients did not initiate ART within one year of their ART eligibility [12]. While younger age, male sex, higher CD4 count, no formal education, and being employed have been implicated as predictors of delay, there is not yet consensus about risk factors for delayed ART initiation [5, 9–12].

Here we report the proportion of HIV-seropositive patients with a CD4 count at or below 350 cells/mm^3^ who do not start ART within two years of entry to care during the study period between 2003 and 2012, using data from the Centers for AIDS Research Network of Integrated Clinical Systems (CNICS). Though HIV/AIDS clinical guidelines did not recommend ART initiation for all HIV-seropositive patients during this study period, treatment for individuals with a CD4 count at or below 350 cells/mm^3^ shifted from a moderate to strong recommendation over this time period [13, 14]. We further compare characteristics at care entry of HIV-seropositive patients who had initiation delayed to those who did initiate ART within two years of care entry. We hypothesized that characteristics associated with lack of adherence to ART, such as younger age and a history of injection drug use, would be associated with delay of ART initiation among those with a strong to moderate recommendation for treatment.

## Materials and methods

### Study sample

CNICS is designed to support population-based HIV research through a comprehensive, standardized clinical data repository from point-of-care electronic medical records. CNICS includes clinical data from over 30,000 HIV-seropositive adults, aged 18 years and older, who initiated primary care after January 1, 1995 at any of eight CNICS sites in the US. These sites include Case Western Reserve University, Fenway Community Health Center of Harvard University, Johns Hopkins University, University of Alabama at Birmingham, University of California at San Diego, University of California at San Francisco, University of North Carolina, and University of Washington. Institutional review boards at each site approved study participation. Participants provided written informed consent or a waiver of informed consent to use clinical and administrative data, as approved by the local institutional review board. CNICS maintains data on patient demographics, disease diagnoses, laboratory data, medication data, health care utilization, vital status, patient reported outcomes, and antiretroviral drug resistance. Historical data on patients’ prior ART and diagnosis history are collected at the time of enrollment, which is at the time of their second clinic visit. Mortality is confirmed from clinic sources, death certificates, and the US Social Security Death Index, which is regularly checked by CNICS sites. Follow-up data are acquired at clinic visits, which are approximately every three months on average.

In this prospective cohort study, the analysis was restricted to HIV-seropositive patients with a baseline CD4 count at or below 350 cells/mm^3^, enrolled in CNICS between January 1, 2003 and December 31, 2012, and who had not initiated ART prior to their CNICS enrollment. Overall, 6,083 patients met these study eligibility criteria. There were 861 patients (14%) excluded due to missing data at baseline including missing HIV-1 RNA viral load (n = 147, 2%), race/ethnicity (n = 588, 10%), and HIV risk factors such as reported injection drug use (n = 155, 3%), and reported male-to-male sexual contact (n = 125, 2%). Patients with a viral load measurement of less than 75 copies/mL indicative of suppressed or undetectable viral load (n = 315, 5%) were excluded because many of these patients may have been misclassified as ART-naïve. Patient characteristics among those excluded followed a similar distribution to those included in the final study sample. This resulted in a final study sample of 4,907 patients.

### Outcome definition

The primary outcome, delayed ART initiation, was defined as failure to initiate a regimen of three or more antiretroviral drugs within the first two years of CNICS enrollment. The date of ART initiation was determined to be the first day in which each patient was prescribed three or more antiretroviral drugs.

### Patient characteristics

The following factors were assessed as predictors of delayed ART initiation: age (18–29, 30–34, 35–39, 40–44, 45–49, and 50–93 years), gender (male/female), race/ethnicity (white non-Hispanic, black non-Hispanic, other non-Hispanic, and Hispanic), enrollment date (in years), HIV transmission risk factor of injection drug use (yes/no) and male-to-male sexual contact (yes/no), AIDS diagnosis (yes/no), HIV-1 RNA viral load (75–9,999, 10,000–99,999, and 100,000–1.6x10^6^ copies/mL), and CD4 cell count (0–199 and 200–350 cells/mm^3^). Age, injection drug use, male-to-male sexual contact, calendar year, and AIDS diagnosis were defined at study enrollment. HIV-1 RNA viral load and CD4 cell count were defined as the most recent lab measurement recorded within 90 days prior to 30 days after study enrollment.

### Statistical analysis

Factors were summarized using percent or median with interquartile range (IQR), as appropriate, according to ART initiation status, lost-to-follow-up, and deaths. Patients were defined as lost to follow-up at one year since their last clinic visit without a recorded lab measurement. Stabilized inverse probability of censoring weights were constructed using two logistic regression models. The first model estimated the unconditional probability of being censored and the second model estimated the probability of being censored conditional on all measured factors. The final weight was the marginal probability of remaining under follow-up divided by the patient’s conditional probability of remaining under follow-up. The stabilized inverse probability of censoring weights had a mean of 1.00 and ranged from 0.95 to 1.52. A weighted log-binomial model was used to estimate crude and multivariable-adjusted risk ratios (RR) comparing delayed ART initiation within two years between patients with and without each risk factor. Confidence intervals were based on the robust variance estimator. Deaths within the two-year ART initiation period were classified based on whether patients did or did not initiate ART before their occurrence of death. Patients initiating ART prior to being lost to follow up within the two-year ART initiation period were classified as initiating ART. The final contrast compares those who fail to initiate ART to those who initiate ART within the two-year period, with patients lost to follow up prior to initiating ART contributing to the weighted model through inverse probability of censoring weights. In multivariable analyses, the RR and 95% CIs were estimated for each factor while adjusting for all other factors.

We categorized age, CD4 cell count, and viral load to relax linearity assumptions. Previous DHHS guidelines suggest that these categories for CD4 cell count and viral load are appropriate cut points for clinical decisions regarding the initiation of therapy among ART-naïve patients [13, 14]. We assessed potential differences by calendar time in the association between risk factors and delayed ART initiation through examination of stratified results by two enrollment date periods. We conducted two sensitivity analyses comparing results with the exclusion of decedents over the two-year risk period and then further adjusting for both clinic site and the number of clinic visits. Data analyses were performed using SAS software version 9.3 (SAS Institute, Inc., Cary, NC).

## Results

The distribution of demographic characteristics for the sample at CNICS enrollment is displayed in Table 1. The majority of patients were men (83%; n = 4,084) and the prevalence of male-to-male sexual contact was 60% (n = 2,925). Thirteen percent (n = 654) of patients reported injection drug use and 29% (n = 1,432) of patients had a prevalent AIDS diagnosis at their CNICS enrollment. The median CNICS enrollment year was 2007 (IQR: 2005, 2010) and 18% of patients were at least 50 years of age. Forty-two percent of patients had a viral load ≥100,000 copies/mL and 59% of patients had a CD4 cell count below 200 cells/mm^3^. The most common racial groups were white non-Hispanic at 41%, black non-Hispanic at 40%, and Hispanic at 13%.

**Table 1:** Characteristics of HIV-seropositive Patients Enrolled in CNICS, 2003–2012.

Characteristic	Non-Initiators			Deaths			Lost to Follow-Up		ART Initiators		Overall	
	(n = 796)			(n = 303)			(n = 930)		(n = 2878)		(n = 4907)	
	N		%	N	%		N	%	N	%	N	%
Enrollment Year^a^	2007 (2005, 2009)			2006 (2004, 2009)			2007 (2005, 2009)		2007 (2005, 2010)		2007 (2005, 2010)	
Gender Women	156		19.6	52	17.2		158	17.0	457	15.9	823	16.8
Age (years) <30	135		17.0	24	8.0		129	13.9	516	17.9	804	16.4
30–34	100		12.6	36	11.9		137	14.7	423	14.7	696	14.2
35–39	137		17.2	44	14.5		156	16.8	507	17.6	844	17.2
40–44	141		17.7	48	15.8		199	21.4	523	18.2	911	18.6
45–49	137		17.2	59	19.5		153	16.5	443	15.4	792	16.1
50+	146		18.3	92	30.4		156	16.8	466	16.2	860	17.5
Race/Ethnicity												
White Non-Hispanic	298		37.4	112	37.0		370	39.8	1210	42.0	1990	40.6
Black Non-Hispanic	358		45.0	139	45.9		405	43.6	1040	36.1	1942	39.6
Other Non-Hispanic	46		5.8	15	5.0		53	5.7	211	7.3	325	6.6
Hispanic	94		11.8	37	12.2		102	11.0	417	14.5	650	13.3
Injection Drug Use Yes	113		14.2	61	20.1		179	19.3	301	10.5	654	13.3
MSM Yes	441		55.4	129	42. 6		553	59.5	1802	62.6	2925	59.6
CD4 Count (cells/mm^3^) <200	393		49.4	241	79.5		510	54.8	1748	60.7	2892	58.9
Viral Load (copies/mL) 100,000+	257		32.3	151	49.8		303	32.6	1340	45.6	2051	41.8
10,000–99,999	311		39.1	110	36.3		423	45.5	1061	36.9	1905	38.8
<10,000	228		28.6	42	13.9		204	21.9	477	16.6	951	19.4
AIDS Diagnosis Yes	230		28.9	141	46.5		257	27.6	804	27.9	1432	29.2

Abbreviations: CNICS, CFAR Network of Integrated Clinical Systems; MSM, ever male-to-male sexual contact; AIDS, acquired immunodeficiency syndrome

^a^Median (interquartile range)

In their first two years of follow-up, 16% (n = 796) of the 4,907 patients did not initiate ART, 6% (n = 303) died, 19% (n = 930) were lost to follow-up, and 59% (n = 2,878) initiated ART. Excluding only patients who were alive and had not initiated ART prior to becoming lost to follow-up (n = 293), the proportion that did not initiate ART within 6 months, 1 year, and 2 years was 31% (n = 1,439), 26% (n = 1,178), and 21% (n = 959), respectively.

Table 2 presents the primary analysis of estimates of the crude and adjusted risk ratios for delayed ART, comparing failure to initiate ART within two years of enrollment for each demographic factor. Women and those reporting injection drug use at enrollment were less likely to initiate ART within two years following CNICS enrollment compared to men (crude RR = 1.28; 95% CI: 1.07, 1.54) and those that did not report injection drug use (crude RR = 1.27; 95% CI: 1.03, 1.56), respectively. After adjustment for other factors, an earlier CNICS enrollment date was the only demographic factor associated with delayed ART initiation. For example, for each one-year increase in CNICS enrollment date, patients were 0.94 times (95% CI: 0.92, 0.97) as likely to delay ART initiation. Age, gender, race/ethnicity, reported injection drug use, and an HIV risk factor of male-to-male sexual contact were not associated with delayed ART initiation after adjustment for other factors.

**Table 2:** Associations between risk factors and lack of ART initiation among HIV-seropositive patients with CD4 at or below 350 cells/mm^3^ enrolled in CNICS (including deaths) with inverse probability of censoring weighting, 2003–2012.

Characteristic	Non-Initiators		ART Initiators		Crude RR^a^ (95% CI)	Adjusted RR^a^ (95% CI)
	(n = 959)		(n = 3655)			
	N	%	N	%		
Enrollment Year^b^	2007 (2005, 2009)		2007 (2005, 2010)		0.94 (0.92, 0.97)	0.94 (0.92, 0.97)
Gender Men	772	80.5	3080	84.3	1	1
Women	187	19.5	575	15.7	1.28 (1.07, 1.54)	0.95 (0.75, 1.21)
Age (years) <30	147	15.3	609	16.7	1	1
30–34	122	12.7	534	14.6	0.96 (0.74, 1.25)	1.01 (0.76, 1.35)
35–39	161	16.8	640	17.5	1.06 (0.83, 1.36)	1.06 (0.81, 1.40)
40–44	166	17.3	676	18.5	1.03 (0.81, 1.33)	0.95 (0.74, 1.24)
45–49	165	17.2	580	15.9	1.19 (0.93, 1.53)	1.13 (0.85, 1.49)
50+	198	20.7	616	16.9	1.34 (1.05, 1.70)	1.25 (0.95, 1.63)
Race/Ethnicity White Non-Hispanic	362	37.8	1522	41.6	1		1	
Black Non-Hispanic	436	45.5	1373	37.6	1.34 (1.14, 1.57)	1.15 (0.96, 1.38)
Other Non-Hispanic	50	5.2	260	7.1	0.82 (0.59, 1.13)	0.80 (0.57, 1.14)
Hispanic	111	11.6	500	13.7	0.94 (0.75, 1.19)	0.93 (0.72, 1.20)
Injection Drug Use No	817	85.2	3208	87.8	1	1
Yes	142	14.8	447	12.2	1.27 (1.03, 1.56)	1.03 (0.81, 1.30)
MSM No	448	46.7	1410	38.6	1	1
Yes	511	53.3	2245	61.4	0.72 (0.62, 0.83)	0.82 (0.68, 1.02)
CD4 Count (cells/mm^3^) <200	524	54.6	2240	61.3	1	1
200–350	435	45.4	1415	38.7	1.33 (1.15, 1.53)	1.31 (1.11, 1.56)
Viral Load (copies/mL) 100,000+	337	35.1	1642	44.9	1	1
10,000–99,999	370	38.6	1414	38.7	1.28 (1.09, 1.51)	1.23 (1.03, 1.47)
<10,000	252	26.3	599	16.4	2.06 (1.71, 2.49)	1.89 (1.53, 2.33)
AIDS Diagnosis No	655	68.3	2609	71.4	1	1
Yes	304	31.7	1046	28.6	1.16 (1.00, 1.36)	1.23 (1.04, 1.47)

Abbreviations: CNICS, CFAR Network of Integrated Clinical Systems; MSM, ever male-to-male sexual contact; AIDS, acquired immunodeficiency syndrome; CI, confidence interval; RR, risk ratio

^a^IP weighted estimates

^b^Median (interquartile range)

Clinical factors, each assessed at enrollment, were examined as to their association with delayed ART after adjustment for all other clinical and demographics factors. As shown in Table 2, clinical factors associated with delayed ART included higher CD4 cell count, lower viral load, and a prevalent AIDS diagnosis. For example, those with CD4 count at least 200 cells/mm^3^ at enrollment were 1.31 (95% CI: 1.11, 1.56) times as likely to delay ART initiation compared to those with CD4 count less than 200 cells/mm^3^. Those with viral load less than 10,000 copies/ml at enrollment were more likely to delay ART initiation compared to those with viral load at least 100,000 copies/ml (adjusted RR = 1.89; 95% CI: 1.53, 2.33). Finally, those with a prevalent AIDS diagnosis at enrollment were more likely to delay ART initiation (adjusted RR = 1.23; 95% CI: 1.04, 1.47).

Table 3 presents estimates of the crude and adjusted risk ratios for delayed ART by CD4 count, a prevalent AIDS diagnosis, and viral load over two enrollment date periods. The association with delayed ART for both baseline CD4 count and a prevalent AIDS diagnosis dissipated in the latter time period. For example, from 2003–2007, those with CD4 count at least 200 cells/mm^3^ at enrollment were 1.59 (95% CI: 1.28, 1.97) times as likely to delay ART initiation compared to those with CD4 count less than 200 cells/mm^3^. However from 2008–2012, those with CD4 count at least 200 cells/mm^3^ at enrollment were 1.04 (95% CI: 0.82, 1.32) times as likely to delay ART initiation compared to those with CD4 count less than 200 cells/mm^3^. The association between viral load and delayed ART initiation was attenuated, but still persisted in the latter time period. In additional sensitivity analyses, excluding decedents over the two-year risk period and further adjusting for both clinic site and the number of clinic visits resulted in no appreciable changes in the patterns seen in the data.

**Table 3:** Association between clinical characteristics and lack of ART initiation across two enrollment periods among HIV-seropositive patients enrolled in CNICS (including deaths) with inverse probability of censoring weighting, 2003–2012.

Enrollment Periods	Characteristic	Non-Initiators (n = 959)		ART Initiators (n = 3655)		Crude RR^a^ (95% CI)	Adjusted RR^a^ (95% CI)
		N	%	N	%		
2003–2007						
	CD4 Count (cells/mm^3^) <200	306	54.1	1247	65.4	1	1
	200–350	260	45.9	659	34.6	1.62 (1.34, 1.96)	1.59 (1.28, 1.97)
	AIDS Diagnosis No	364	64.3	1286	67.5	1	1
	Yes	202	35.7	620	32.5	1.16 (0.95, 1.41)	1.43 (1.15, 1.77)
	Viral Load (copies/mL) 100,000+	211	37.3	921	48.3	1	1
	10,000–99,999	208	36.8	717	37.6	1.28 (1.03, 1.58)	1.20 (0.96, 1.50)
	<10,000	147	26.0	268	14.1	2.39 (1.86, 3.07)	2.08 (1.59, 2.71)
2008–2012							
	CD4 Count (cells/mm^3^) <200	218	55.5	993	56.8	1	1
	200–350	175	44.5	756	43.2	1.06 (0.85, 1.32)	1.04 (0.82, 1.32)
	AIDS Diagnosis No	291	74.1	1323	75.6	1	1
	Yes	102	26.0	426	24.4	1.09 (0.85, 1.41)	1.03 (0.80, 1.34)
	Viral Load (copies/mL) 100,000+	126	32.1	721	41.2	1	1
	10,000–99,999	162	41.2	697	39.9	1.32 (1.03, 1.71)	1.32 (1.01, 1.72)
	<10,000	105	26.7	331	18.9	1.82 (1.36, 2.44)	1.72 (1.27, 2.32)

Abbreviations: CNICS, CFAR Network of Integrated Clinical Systems; CI, confidence interval; RR, risk ratio

^a^IP weighted estimates

## Discussion

We found that among ART-naïve patients with CD4 count at or below 350 cells/mm^3^, higher CD4 count, lower viral load, a prevalent AIDS diagnosis, and an earlier CNICS enrollment date were each independently associated with failure to initiate ART within the first two years after enrollment in CNICS between January 1, 2003 and October 1, 2012. Lower viral load was the sole clinical factor to remain associated with failure to initiate ART over the entire study period. Female gender, older age, black race/ethnicity, reported injection drug use, and lack of reported male-to-male sexual contact were demographic characteristics associated with delayed ART initiation in the unadjusted analysis, but did these associations did not persist after adjustment for other demographic and clinical characteristics.

The fact that entering CNICS with a CD4 count of at least 200 cells/mm^3^ was associated with delayed ART initiation compared to those with CD4 count less than 200 cells/mm^3^ was somewhat anticipated, but strength of this association given the extent of the delay (two years) was greater than expected. Clinical guidelines for ART initiation among treatment-naïve HIV patients have evolved. In 2003, at the beginning of study follow-up, ART initiation was recommended for HIV-seropositive individuals with a CD4 count less than 200 cells/mm^3^ [13]. However the strength of recommendation for ART initiation among asymptomatic patients with a CD4 count at least 200 cells/mm^3^ was carefully evaluated at the discretion of the patient and physician, with regard to weighing potential benefits and risks of early or delayed therapy initiation, especially among those patients with a CD4 count greater than 350 cells/mm^3^ for whom the benefits of ART initiation were uncertain [13]. By 2007, guidelines recommended ART initiation for those with CD4 count less than 350 cells/mm^3^ [14]. Our findings that an earlier year of enrollment and higher CD4 count are associated with failure to initiate ART, and that the association between higher CD4 count and delayed ART is attenuated over time likely reflect these changes in the HIV/AIDS clinical guidelines and treatment patterns regarding treatment initiation. Though guidelines also recommended ART initiation for patients with an AIDS diagnosis, it is possible that our findings that a prevalent AIDS diagnosis was associated with delayed ART initiation could have resulted from a lower engagement in care among these patients. Patients with a prevalent AIDS diagnoses were more likely to receive their AIDS diagnosis earlier in the HIV epidemic and prior to their CNICS enrollment date. It is possible these patients may have received care but were not consistently engaged in care.

We know of no other studies in US populations investigating the proportion of patients, for whom treatment was strongly to moderately recommended, with delayed ART initiation and their characteristics. Given that clinical guidelines recommended ART initiation for all patients regardless of CD4 count as of March 2012, it has become more important to characterize which patients are eligible for increased benefits of treatment initiation. A study of HIV patients newly clinically eligible for ART through their first reported CD4 count <350 cells or an AIDS-defining illness from the NA-ACCORD between 2001 and 2009 assessed factors associated with timely ART initiation [15]. Increasing age, later calendar year, male sex, a lack of history of injection drug use, a lower number of psychosocial barriers, lower CD4 count, increasing viral load, and lack of incident AIDS-defining illness were associated with a shorter time to ART initiation [15]. They also found that none of these characteristics varied over time in their association with a shorter time to ART initiation [15]. Our findings that some of these characteristics are associated with delayed ART initiation within a two-year period are consistent with their findings. However, our findings also suggest that the importance of clinical characteristics associated with delayed ART initiation may be diminishing over time, within the exception of lower viral load.

It was unexpected that race/ethnicity, gender, and a history of injection drug use were not associated with delayed ART initiation in the adjusted analysis. During this study period, concerns about the role of treatment adherence in treatment effectiveness played a pivotal role in deciding whether to initiate treatment in ART-naïve patients [13, 14]. Previous studies have found that blacks and women experience less time on ART while in HIV care compared to whites and men, and that older age and male sex are associated with a shorter time to ART initiation [15, 16]. A recent study of patients from the Johns Hopkins HIV Clinical Cohort compared differences in the average time for each stage of the HIV care continuum between people who inject drugs and those who do not across 10 years following their enrollment [17]. Patients reporting a history of injection drug use spent an additional 5.5 months in HIV care but not on ART, and an additional 5.0 months on ART but not virally suppressed compared to those who did not have an injection drug use history [17]. Given these findings, the disparities between gender, race, and history of injection drug use in access and barriers to care, and the potential concerns regarding adherence to treatment, it is expected that these demographic characteristics are associated with two-year delayed ART initiation as shown in the unadjusted analysis. However, our results suggest that these demographic differences may be explained by unequal distributions of adverse clinical characteristics, which appear to be stronger risk factors for treatment initiation. For example, the strength of the association between non-Hispanic black race/ethnicity and delayed ART initiation was attenuated after adjustment for other factors.

The present work has several limitations. First, this is an observational study and therefore estimates are subject to possible confounding bias. Multiple adjusted effects from a single model should be interpreted with caution, and the potential for overadjustment bias, as well as adjusting for unnecessary variables should not be overlooked as these adjustments can result in increased bias or reduced precision [18, 19]. However the primary focus of this research was to describe common characteristics of individuals not receiving prompt treatment with the goal of improving HIV care.

Second, it is assumed measurements were taken with negligible error. CNICS is a clinic-based research network using point-of-care data collection to deliver medical information suited for research in a real-time setting using its standardized clinical repository. Given the standardized procedures in data collection, misclassification is likely to be modest. However, there could be unmeasured misclassification of clinical factors, including CD4 count and viral load, where it is possible that those patients classified as treatment-naïve could have previously received treatment. Third, results pertain to patients enrolled in HIV care during this study period from 2003–2012, though treatment patterns and guidelines have continued to evolve over time. Fourth, though not included in this study, insurance status and socioeconomic status are other patient characteristics that could be considered as potential risk factors for delayed ART initiation.

Lastly, deaths over the two-year time period were relatively infrequent, but losses to follow up were not. Inverse probability of censoring weights were used and we assume that the data are missing at random such that covariate information in the data can be used to eliminate selection bias induced by censoring individuals lost to follow up prior to therapy initiation.

A major strength of the CNICS cohort is that it is a large multicenter HIV clinical cohort that has a standardized clinical data repository based upon prospective collection of comprehensive patient data. Given this strong research network, there was not a substantial amount of missing data for any of the covariates within this study. However, the general population is likely to include HIV-seropositive patients who are more difficult to enroll in clinical cohorts, such as those individuals with lower access and engagement in the healthcare system. Our results estimating the proportion of delayed ART initiation are likely to be conservative relative to other US HIV-seropositive patient populations. Future studies can help to identify whether these same characteristics remain consistent risk factors for delayed ART initiation among other HIV-seropositive patient populations.

## Conclusions

This study identified characteristics of patients who delay ART initiation beyond the first two years of entering in care, which included an earlier CNICS enrollment date, higher CD4 count, lower viral load, and a prevalent AIDS diagnosis. This prolonged delay in ART initiation among patients with a moderate to strong recommendation under past guidelines suggests that treatment practice under current guidelines recommending therapy initiation to all HIV-seropositive patients should be carefully scrutinized. The tendency to delay therapy in those patients with a lower viral load may still persist despite clear evidence of the benefits of immediate therapy initiation [20]. These clinical characteristics may continue to be important with current ART guidelines in providing targets for closer monitoring and intervention to reduce disparities in HIV care.

## Supporting information

S1 Table Associations between risk factors and lack of ART initiation among HIV-seropositive patients with CD4 below 200 cells/mm^3^ enrolled in CNICS (including deaths) with inverse probability of censoring weighting, 2003–2012. (DOCX)
