# Supplementary material for: Risk factors for delayed antiretroviral therapy initiation among HIV-seropositive patients
Source: PLoS One. 2017 Jul 10;12(7):e0180843. doi: 10.1371/journal.pone.0180843 (PMC5507276; doi:10.1371/journal.pone.0180843)
Supplement: S1 Table — (DOCX) [file pone.0180843.s001.docx]

**S1 Table. Associations between risk factors and lack of ART initiation among HIV-seropositive patients with CD4 below 200 cells/mm^3^ enrolled in CNICS (including deaths) with inverse probability of censoring weighting, 2003-2012.**

|  |  | | | | Non-Initiators | | | ART Initiators | |  | |  | |
| --- | --- | --- | --- | --- | --- | --- | --- | --- | --- | --- | --- | --- | --- |
|  | Characteristic | | | | (n=524) | | | (n=2240) | | Crude RR^a^ | | Adjusted RR^a^ | |
|  |  | | | | N | | % | N | % | (95% CI) | | (95% CI) | |
|  | Enrollment Year^b^ | | | | 2007 (2005, 2009) | | | 2007 (2005, 2009) | | 0.97 (0.94, 1.00) | | 0.98 (0.94, 1.01) | |
|  | Gender | Men | | | 421 | 80.3 | | 1886 | 84.2 | 1 | | 1 | |
|  | Women | | | | 103 | 19.7 | | 354 | 15.8 | 1.30 (1.02, 1.66) | | 1.00 (0.73, 1.36) | |
|  | Age (years) | <30 | | | 57 | 10.9 | | 282 | 12.6 | 1 | | 1 | |
|  | 30-34 | | | | 63 | 12.0 | | 321 | 14.3 | 0.97 (0.66, 1.44) | | 0.94 (0.61, 1.44) | |
|  | 35-39 | | | | 76 | 14.5 | | 412 | 18.4 | 0.92 (0.63, 1.34) | | 0.90 (0.60, 1.34) | |
|  | 40-44 | | | | 99 | 18.9 | | 448 | 20.0 | 1.11 (0.77, 1.59) | | 0.93 (0.63, 1.38) | |
|  | 45-49 | | | | 100 | 19.1 | | 385 | 17.2 | 1.29 (0.90, 1.85) | | 1.15 (0.77, 1.71) | |
|  | 50+ | | | | 129 | 24.6 | | 392 | 17.5 | 1.62 (1.15, 2.30) | | 1.36 (0.93, 2.00) | |
|  | Race/Ethnicity | White Non-Hispanic | | | 194 | 37.0 | | 869 | 38.8 | 1 |  | 1 |  |
|  | Black Non-Hispanic | | | | 255 | 48.7 | | 903 | 40.3 | 1.27 (1.03, 1.57) | | 1.04 (0.82, 1.33) | |
|  | Other Non-Hispanic | | | | 19 | 3.6 | | 158 | 7.1 | 0.54 (0.33, 0.89) | | 0.49 (0.29, 0.85) | |
|  | Hispanic | | | | 56 | 10.7 | | 310 | 13.8 | 0.81 (0.59, 1.13) | | 0.83 (0.59, 1.18) | |
|  | Injection Drug Use | | | No | 439 | 83.8 | | 1951 | 87.1 | 1 | | 1 | |
|  | Yes | | | | 85 | 16.2 | | 289 | 12.9 | 1.31 (1.00, 1.70) | | 1.05 (0.78, 1.42) | |
|  | MSM | | | No | 272 | 51.9 | | 967 | 43.2 | 1 | | 1 | |
|  | Yes | | | | 252 | 48.1 | | 1273 | 56.8 | 0.70 (0.58, 0.85) | | 0.87 (0.67, 1.13) | |
|  | CD4 Count (cells/mm^3^) | | | <100 | 322 | 61.5 | | 1403 | 62.6 | 1 | | 1 | |
|  | 100-199 | | | | 202 | 38.6 | | 837 | 37.4 | 1.05 (0.87, 1.28) | | 1.09 (0.88, 1.37) | |
|  | Viral Load (copies/mL) | | | 100,000+ | 243 | 46.4 | | 1232 | 55.0 | 1 | | 1 | |
|  | 10,000-99,999 | | | | 177 | 33.8 | | 754 | 33.7 | 1.19 (0.96, 1.47) | | 1.12 (0.89, 1.41) | |
|  | <10,000 | | | | 104 | 19.9 | | 254 | 11.3 | 2.07 (1.59, 2.70) | | 1.99 (1.48, 2.69) | |
|  | AIDS Diagnosis | | No | | 283 | 54.0 | | 1367 | 61.0 | 1 | | 1 | |
|  | Yes | | | | 241 | 46.0 | | 873 | 39.0 | 1.35 (1.11, 1.63) | | 1.35 (1.09, 1.67) | |

Abbreviations: CNICS, CFAR Network of Integrated Clinical Systems; MSM, ever male-to-male sexual contact; AIDS, acquired immunodeficiency syndrome; CI, confidence interval; RR, risk ratio

^a^IP weighted estimates

^b^Median (interquartile range)
